# Blind Image Restoration Enhances Digital Autoradiographic Imaging of Radiopharmaceutical Tissue Distribution

**DOI:** 10.2967/jnumed.121.262270

**Published:** 2022-04

**Authors:** Lu Peng, Benabdallah Nadia, Jiang Wen, Brian W. Simons, Zhang Hanwen, Robert F. Hobbs, Ulmert David, Brian C. Baumann, Russell K. Pachynski, Abhinav K. Jha, Daniel L.J. Thorek

**Affiliations:** 1 Department of Biomedical Engineering, Washington University in St. Louis, St. Louis, Missouri;; 2 Department of Radiology, Mallinckrodt Institute of Radiology, Washington University School of Medicine, St. Louis, Missouri;; 3 Program in Quantitative Molecular Therapeutics, Washington University School of Medicine, St. Louis, Missouri;; 4 Department of Biomedical Engineering, Johns Hopkins University, Baltimore, Maryland;; 5 Center for Comparative Medicine, Baylor College of Medicine, Houston, Texas;; 6 Radiation Oncology and Molecular Radiation Sciences, Johns Hopkins University School of Medicine, Baltimore, Maryland;; 7 Department of Molecular and Medical Pharmacology, UCLA, Los Angeles, California;; 8 Ahmanson Translational Imaging Division, David Geffen School of Medicine, UCLA, Los Angeles, California;; 9 Department of Radiation Oncology, Washington University School of Medicine, St. Louis, Missouri;; 10 Division of Oncology, Washington University School of Medicine, St. Louis, Missouri; and; 11 Oncologic Imaging Program, Siteman Cancer Center, Washington University School of Medicine, St. Louis, Missouri

**Keywords:** digital autoradiography, blind image restoration, Poisson–gaussian noise model, positron, α-particle emission

## Abstract

Digital autoradiography (DAR) is a powerful tool to quantitatively determine the distribution of a radiopharmaceutical within a tissue section and is widely used in drug discovery and development. However, the low image resolution and significant background noise can result in poor correlation, even errors, between radiotracer distribution, anatomic structure, and molecular expression profiles. Differing from conventional optical systems, the point-spread function in DAR is determined by properties of radioisotope decay, phosphor, and digitizer. Calibration of an experimental point-spread function a priori is difficult, prone to error, and impractical. We have developed a content-adaptive restoration algorithm to address these problems. **Methods:** We model the DAR imaging process using a mixed Poisson–gaussian model and blindly restore the image by a penalized maximum-likelihood expectation-maximization algorithm (PG-PEM). PG-PEM implements a patch-based estimation algorithm with density-based spatial clustering of applications with noise to estimate noise parameters and uses L2 and Hessian Frebonius norms as regularization functions to improve performance. **Results:** First, PG-PEM outperformed other restoration algorithms at the denoising task (*P <* 0.01). Next, we implemented PG-PEM on preclinical DAR images (^18^F-FDG, treated mouse tumor and heart; ^18^F-NaF, treated mouse femur) and clinical DAR images (bone biopsy sections from ^223^RaCl_2_-treated castration-resistant prostate cancer patients). DAR images restored by PG-PEM of all samples achieved a significantly higher effective resolution and contrast-to-noise ratio and a lower SD of background (*P <* 0.0001). Additionally, by comparing the registration results between the clinical DAR images and the segmented bone masks from the corresponding histologic images, we found that the radiopharmaceutical distribution was significantly improved (*P <* 0.0001). **Conclusion:** PG-PEM is able to increase resolution and contrast while robustly accounting for DAR noise and demonstrates the capacity to be widely implemented to improve preclinical and clinical DAR imaging of radiopharmaceutical distribution.

## Visual Abstract

**Figure:**
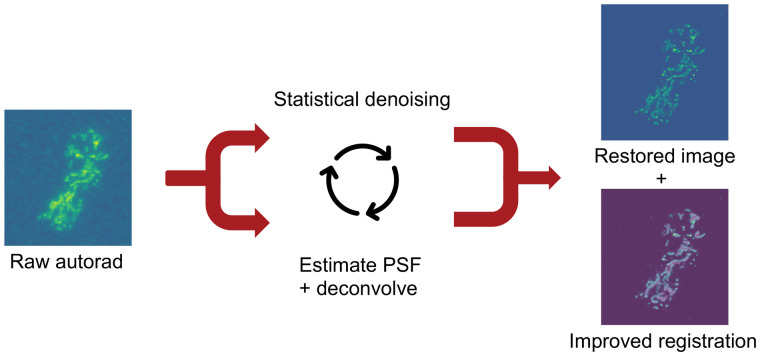

Autoradiography is a powerful, high-resolution, and quantitative molecular imaging technique used to study the tissue distribution of radioisotopes in biologic systems and for analytic assays (*1*–*4*). Originally, radioactivity distributions were acquired using photographic emulsions, which are of high resolution but require time-consuming, fickle, and variable processes. Currently, phosphor imaging plate–based digital autoradiography (DAR) has supplanted film because of its linear activity response, nondestructive approach, lack of a chemical-processing requirement, large dynamic range, and considerable sensitivity (*2*,*4*,*5*).

Generally, DAR is performed by placing tissue samples containing radioactivity apposed to the phosphor screen, which absorbs and stores the energy of the radioactive emissions, creating a latent image of activity distribution (Fig. 1A). Except for very low energy β-emitters (tritium), the phosphor layer and the specimens are typically separated by low-attenuation film to prevent contamination of the screen itself, and exposure lasts hours to days. The phosphor plate is raster-scanned with a small focal-spot red laser, and the photostimulated light is collected by a photomultiplier tube to form a digital image (Fig. 1B). The intensity of emitted light is proportional to the amount of radioactivity in the tissue sample.

**FIGURE 1.**
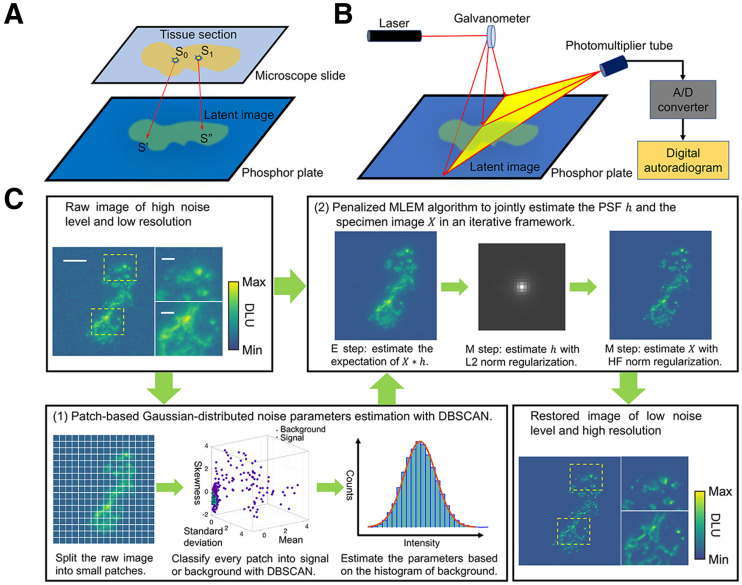
DAR imaging process and PG-PEM algorithmic framework. (A) Latent image generation, in which *S*_0_ and *S*_1_ are 2 point sources, detected at *S′* and *S″*. (B) DAR image generation. (C) PG-PEM framework: noise parameter estimation (1); PSF and specimen image estimation (2). Scale bars: large figure, 2.3 mm; small figures, 0.54 mm. A/D = analog/digital; DBSCAN = density-based spatial clustering of applications with noise; DLU = digital light unit; E = expectation; HF = hessian Frebonius; M = maximization.

Suboptimal image quality in DAR limits assessment of radioligand evaluation. Unlike optical microscopy systems, DAR does not use an aperture or collimator, and the solid angle subtended at the samples by the imaging plate is almost 2π. Therefore, the point-spread function (PSF) results from isotropic emission and is dependent on a combination of energy dispersion in the phosphor, plate properties (lattice and grain size), and readout laser, and physical properties also make the PSF isotope-dependent. Additionally, replicating relevant features of the signal for DAR acquisitions in a phantom is difficult. In aggregate, it is thus not practical to calibrate the PSF beforehand.

Apart from blurring effects caused by PSF, background signal caused by environmental radiation is always present in the imaging process. DAR noise can be attributed to multiple sources: Poisson noise exists in the photon-counting imaging system; gaussian noise comes from the imaging reader readout process, phosphor sheet inhomogeneities, and grain (*6*). Few approaches have been tested to overcome noise and blur-related artifacts: a regularized iteration method after noise filtration (*7*) and the modeling of noise features (*8*). The results from these investigations are not ideal and have not been widely adopted, in part because several have used an emulsion film–based system (the predecessor to phosphor storage plate technology) and noise amplification effects. Common to these approaches are precalibration of PSF by a nonideal resolution phantom.

To model the noise in DAR systems of many isotopes, a blind estimation approach for restoration is preferred. Recently, a mixed-noise model has been used to denoise digital images, which can improve the quality of images contaminated by Poisson and gaussian noise sources (*9*–*12*). A key step in such a model is estimation of noise parameters. For single-image restoration, patch-based (*9*), segmentation-based (*11*), or Fourier-based (*12*) methods have been developed, and several blind and nonblind image restoration techniques for biomedical images have been advanced (*13*–*19*). For the specific task of blind restoration, the regularizations for PSF and specimen are considered in some of these methods, providing a path forward for blind DAR estimation.

Here, a blind image restoration algorithm based on a mixed Poisson–gaussian noise model and a penalized maximum-likelihood expectation maximization (MLEM) algorithm, PG-PEM, is presented. We first describe this model in the context of the DAR imaging process along with a patch-based noise parameter estimation method. We incorporate a penalized MLEM algorithm to jointly estimate the restored specimen image and corresponding PSF. L2 and Hessian Frebonius norms are implemented for PSF and specimen signal separately, to improve the quality of the restored image. PG-PEM improves resolution, improves contrast, and suppresses noise more effectively than contemporary restoration approaches, using both preclinical and clinical applied diagnostic and therapeutic radiopharmaceuticals.

## MATERIALS AND METHODS

### Mouse Tumor, Heart, and Femur Preparation

Experiments were performed in accordance with approved protocols (Institutional Animal Care and Use Committee protocol 2019006). Male C57BL/6 mice (6–10 wk old) from Jackson Laboratory were administered approximately 7.4 MBq (200 μCi) of either ^18^F-FDG or ^18^F-NaF, and harvested at 1 h. Tissues were embedded in optimal-cutting-temperature medium, frozen on dry ice, and sectioned at 8 μm by a cryostat (CM188; Leica). For all radiographic exposures, multisensitive phosphor plates were exposed at −20°C and read as digital light units using a Cyclone Plus (Perkin Elmer). We then used ImageJ (*20*) to crop regions of interest.

### Human Bone Biopsy Preparation

Bone biopsies were obtained from 7 metastatic castration-resistant prostate cancer patients under fluoroscopic guidance after a bone scan, 24 h after injection of ^223^RaCl_2_. The institutional review board approved this study (Human Research Protection Office protocol 201411135), and all subjects provided written informed consent. The biopsy sample was fixed in 4% paraformaldehyde for 24 h, transferred to 30% sucrose for 24 h, frozen, cut, and imaged.

### Staining and Imaging

Sections were stained with hematoxylin and eosin and scanned at ×10 (Nikon Eclipse Ti2 for mouse tumor, heart, and femur slides; Zeiss Axio Scan Z1 for human bone biopsy slides).

### Overview of Image Formation Model and Restoration Algorithm

According to the DAR imaging process, its physical model can be expressed as Equation 1:

$$R_{p}=\text{α}Q_{p}+N_{p},\;Q_{p}∼P\left[{\left(X*h\right)}_{p}+b_{p}\right],\;N_{p}∼N\left(0,{\text{σ}}_{G}^{2}\right),$$ Eq. 1

where *p* is the pixel index, *R* the raw image, α the scaling factor corresponding to the gain of the imaging system, *X* the clean radioactive signal, *h* the PSF, *b* the mean of background, P[x] the Poisson noise with mean x, and $N(0,{\text{σ}}_{G}^{2})$ the gaussian-distributed readout noise with mean of 0 and SD ${\text{σ}}_{G}$. Here, we assume *b_p_* is invariant because of the homogeneous radiation around the tissue.

To estimate *X,* a careful modeling of gaussian noise $N(0,{\text{σ}}_{G}^{2})$ and Poisson noise αP[b] from background *b* is necessary. We implement a noise model to jointly estimate parameters of the 2 components. This is based on the fact that Poisson distribution can be feasibly approximated by a gaussian distribution when *b* is greater than 3 (Supplemental Fig. 1; supplemental materials are available at http://jnm.snmjournals.org) (*21*). Notably, this condition is always satisfied for DAR imaging, and therefore, the 2 independent noise features are summed into a new single gaussian-distributed noise (Supplemental Note 1.1). Consequently, the raw image can be reorganized into a Poisson-distributed signal, $\text{α}P[X_{p}*h]$, and gaussian-distributed noise, $N({\text{μ}}_{N},{\text{σ}}_{N}^{2})$, with mean of α*b* and variance of ${\text{α}}^{2}b+{\text{σ}}_{G}^{2}$. Obviously, $N({\text{μ}}_{N},{\text{σ}}_{N}^{2})$ describes the statistical characterization of the background of DAR images.

Nontissue areas in DAR should have only background and noise and be highly similar to each other. From this assumption, we propose a patch-based estimation algorithm using density-based spatial clustering of applications with noise (Fig. 1C(1); Supplemental Note 1.2 (*22*); Supplemental Algorithm 1; Supplemental Fig. 2) (*23*) to robustly segment background and estimate ${\text{μ}}_{N}$ and ${\text{σ}}_{N}$.

The PG-PEM algorithm uses these noise parameters and the raw image to blindly estimate *X* based on a penalized MLEM algorithm (Fig. 1C(2); Supplemental Notes 1.3 and 1.4 (*24**,* *25*)). The expectation step aims to eliminate the gaussian-distributed noise, $N({\text{μ}}_{N},{\text{σ}}_{N}^{2})$, by calculating the expectation of *X* ∗ *h,* whereas the maximization step deconvolutes the blurry image corrupted by Poisson-distributed data by jointly estimating *h* and *X*. In practice, the blind deconvolution problem is highly ill-posed. Through the iteration process, *h* tends to converge toward a δ-function because of high-frequency noise in the specimen image. To avoid the trivial solution and considering the smooth characteristics of *h,* it is regularized by L2 norm. L2 norm is linearly correlated to the power of *h*. Therefore, the smaller the L2 norm is, the smaller and thus smoother *h* is. During the same process, the noise of the estimated *X* may be amplified. Total variation is a popular approach (*16*,*19*) to suppressing such noise by restraining the summation of the derivative of an image, according to the empiric summary that signals are usually successive whereas noise arises randomly. However, total variation oversharpens boundaries between different regions, generating a staircase effect. To avoid this artifact, we implemented Hessian Frebonius norm regularization to enable smoother transitions between different regions and to suppress noise simultaneously (*15*,*17*,*18*). Compared with total-variation regularization, Hessian Frebonius is a second-order derivative norm and forces the second-order derivative to be sparse. The continuity between different pixels agrees more with the characteristics of biologic autoradiogram data. The regularization strengths for *h* and *X* are controlled by their regularization parameters ${\text{λ}}_{h}$ and ${\text{λ}}_{X}$, respectively.

For our novel PG-PEM, initial estimates for *h* and *X* are needed. The raw image *R* is set as the initial guess of *X* divided by α. *h* can be initialized on the basis of the imaging model. Apart from even scattering, making *h* circularly symmetric, the finite focal point effect of the image reader and the modulation transfer function of the phosphor plate have minor effects on *h*. However, it is unnecessary to build a PSF model accounting for all effects in a blind restoration framework. Instead, initialization of *h* is based on the inverse square law (*26*) when only considering the scattering (Supplemental Note 1.5; Supplemental Fig. 3). Finally, the scaling factor α must be calibrated. Methods previously presented for optical imaging (*11*,*18*) are insufficiently robust for DAR images because it is difficult to find enough homogeneous regions to calibrate α. Empiric calibration is impractical and generally infeasible because of the stochastic decay process and short half-lives in DAR. Fortunately, the mixed Poisson–gaussian data can be approximated as a shifted-Poisson form (*18*), and further, in the deconvolution of Poisson-distributed images, results are not affected by this scaling parameter. Thus, PG-PEM yields a calibration-free algorithm when α is set in a proper range (Supplemental Note 1.6). The detailed algorithmic framework and runtime analysis are summarized (Supplemental Note 1.7; Supplemental Algorithm 2; Supplemental Table 1).

### Quality Metrics

For experiments, the full width at half maximum, the SD of the background (STDB), and the contrast-to-noise ratio (CNR) are set as the accuracy metrics because of the lack of ground truth. Full width at half maximum and STDB can evaluate the resolution and noise level separately, whereas CNR assesses overall performance.

For DAR, it is difficult to measure full width at half maximum using microbeads. Alternatively, we use a recently published decorrelation-based method (Supplemental Fig. 4) (*27*). This method estimates not the theoretic resolution of the imaging system but the highest frequency with sufficiently high signal in relation to noise. We refer to the estimated full width at half maximum as effective resolution.

For simulations, accuracy metrics include root-mean-square error, signal-power–to–noise-power ratio (SNR), and structural similarity (*28*), with which the estimated images can be compared with the ground truth directly. These metrics, along with CNR, are defined in Supplemental Note 2.

### Statistical Analysis

Quantitative data are presented as box-and-whisker plots (center line, median; limits, 75% and 25%; whiskers, maximum and minimum). We used paired 2-sided Student *t* testing to compare the data of raw and PG-PEM–restored DAR images, and we used the paired 1-way analysis of variation to compare all other data (Prism 8; GraphPad Software Inc.).

## RESULTS

### Assessment of Image Enhancement

We benchmarked the performance of several restoration frameworks: Richardson–Lucy (RL) (*13*), RL with wavelet-based residue denoising (RD) (*29*), Shift–Poisson (SP) (*18*), PG-PEM with no regularization for *X* (NP), and PG-PEM with total-variation regularization (TV). For comparison, we have applied our novel background reduction and blind restoration to all approaches and tuned *h* to be similar (Supplemental Notes 3.1–3.5). PG-PEM, together with the 5 modified reference algorithms, was implemented on both simulated images (Supplemental Note 4.1) and experimental images. Regularization parameters are tuned (Supplemental Note 4.2; Supplemental Figs. 5–6), and comparisons on simulated data are analyzed (Supplemental Note 4.3; Supplemental Figs. 7–11).

**FIGURE 2.**
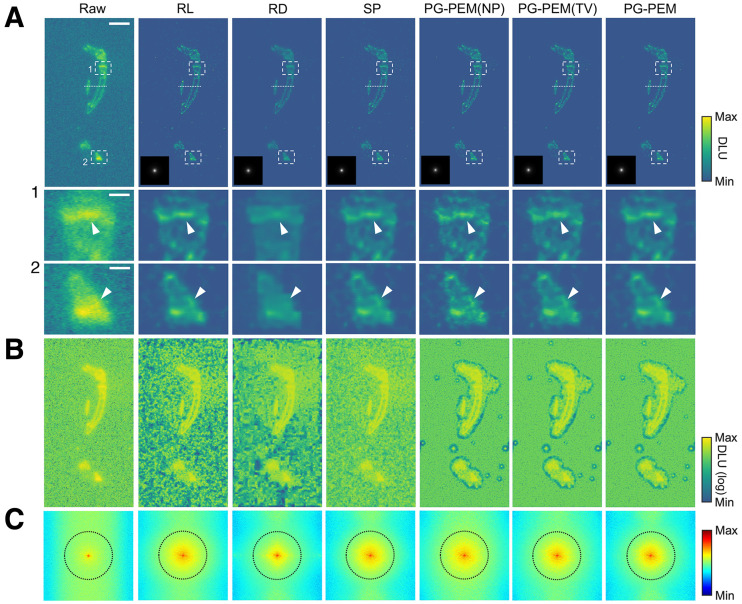
Blind restoration improves DAR. (A) Raw DAR image from mouse hindlimb after ^18^F-NaF PET imaging and its restoration results using modified restoration algorithms. Estimated PSFs are inset in gray scale. (B) Log-scale transformed images from A for background appraisal. (C) Log-scale amplitude of Fourier transform of raw and restored images from A. Scale bars: 4.95 mm (A); 0.86 mm (A1 and A2). DLU = digital light unit.

**FIGURE 3.**
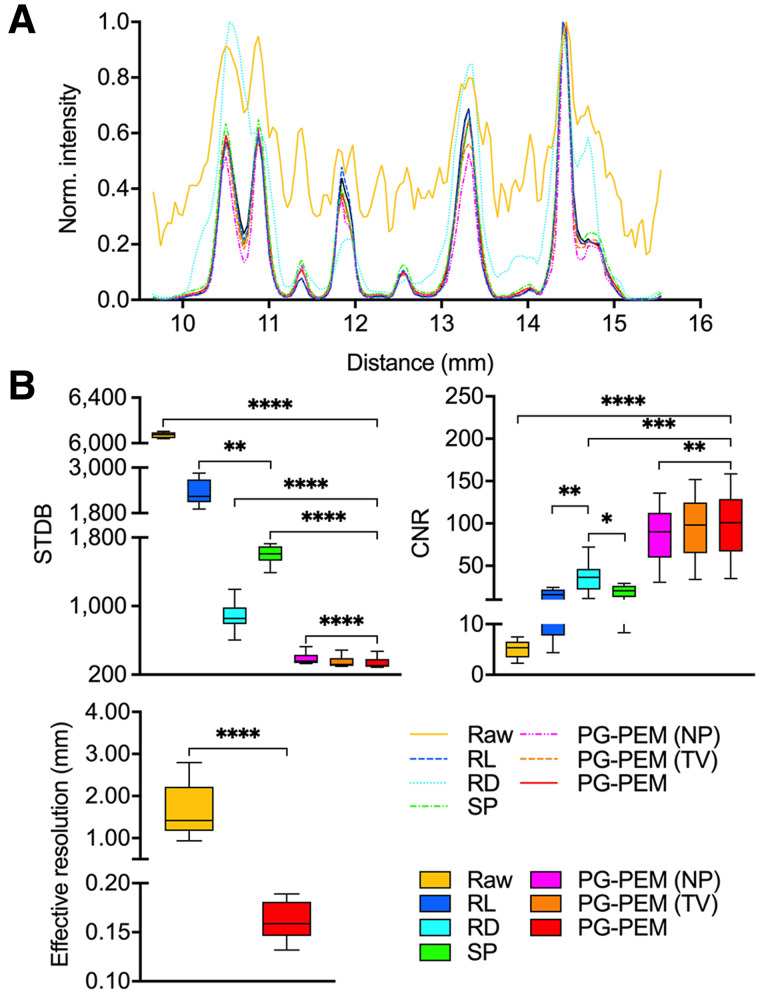
Quantitative assessment of different blind restoration approaches. (A) Profiles of dashed lines in Figure 2A. (B) STDB, CNR, and effective-resolution comparisons of approaches. *P < 0.05. **P < 0.01. ***P < 0.001. ****P < 0.0001.

DAR images (*n* = 10) acquired from the mouse hindlimb after ^18^F-NaF PET imaging were used as experimental data and to evaluate the performance of image restoration approaches. As is standard for short-lived diagnostic radioisotopes and required tissue-processing, sectioning, and exposure times, the SNRs of the raw images are low, providing a model setting for comparison. Visual assessment and analyses (Figs. 2 and 3; Supplemental Fig. 12) show that implementation of restoration algorithms improved resolution and suppressed noise to varying magnitudes. Log-scale images reveal that NP, TV, and PG-PEM have a more homogeneous background than other methods, a result of splitting the image components into Poisson-distributed signal and gaussian-distributed noise. The nonhomogeneous background in RL, RD, and SP correspond to noise and false-positive signal generated in their restoration process.

Next, we assessed the log-scale amplitude of the Fourier space. Because *h* is isotropic, the resolution of DAR images should be at least quasiisotropic. Curiously, we observed that high frequencies tended along the horizontal direction and were highly nonisotropic, which corresponds to the noise. By comparing the frequency maps of NP, TV, and PG-PEM, we found that the nonisotropic components of NP have the highest energy. TV produces a broader nonisotropic frequency portion than PG-PEM and a staircase effect. These, along with STDB and CNR, indicate that PG-PEM is the best denoiser. Meanwhile, RL, SP, NP, TV, and PG-PEM share similar quasiisotropic areas in the dotted black circles (the decorrelation boundaries defined in Fig. 2C), whereas that of RD has the lowest energy. The resolution of RD is the lowest because wavelet-denoising processes remove fine details. With an MLEM restoration framework (and the same regularization strategy for PSF *h*), RL, SP, NP, TV, and PG-PEM share similar resolutions. Notably, because of the lack of a regularization strategy for *X,* the resolution of NP may be slightly higher than those of the other methods, which can be neglected because of the impact of noise. The effective resolution improves at least 5-fold after restoration by PG-PEM (*P <* 0.0001). These data, along with the simulation results, demonstrate that PG-PEM is the best performer for blind restoration of DAR images.

### PG-PEM Improves DAR of Diagnostic Radiopharmaceuticals

**FIGURE 4.**
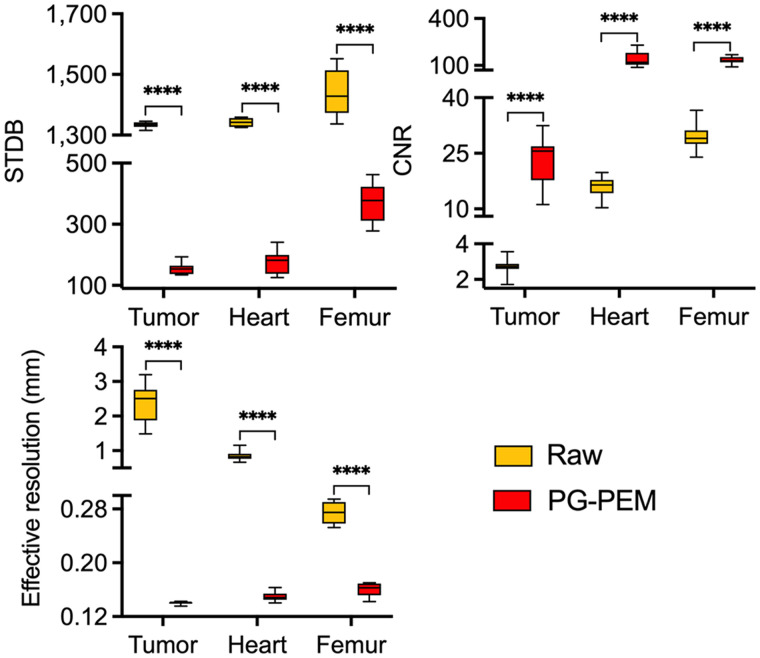
STDB, CNR, and effective resolution assessment of PG-PEM for preclinical DAR images. ****P < 0.0001.

To determine whether PG-PEM could improve the quality of DAR images in diagnostic radiopharmaceuticals, we investigated the distribution of the widely used metabolic tracer ^18^F-FDG, and the bone-seeking ^18^F-NaF, in tissue samples from mouse tumor, heart, and femur (*n* = 10 per group). We used PG-PEM to restore these data, and we calculated STDB, CNR, and effective resolution for comparison to the raw images (Fig. 4). These results demonstrate the image quality improvement after restoration. Notably, a nonglycolytic (prostate) tumor section, which takes up little ^18^F-FDG, has an extremely low SNR. Nevertheless, PG-PEM suppresses background noise and improves the resolution of regions of uptake (Supplemental Fig. 13). RL and SP algorithms were chosen as references to restore the same DAR images from tumors imaged with ^18^F-FDG (Supplemental Fig. 14). Compared with PG-PEM, the results of RL and SP, especially their background components, have more apparent noise. The corresponding STDB and CNR reveal that PG-PEM is superior to restore DAR images under extremely low-SNR conditions, with a *P* value of less than 0.0001.

**FIGURE 5.**
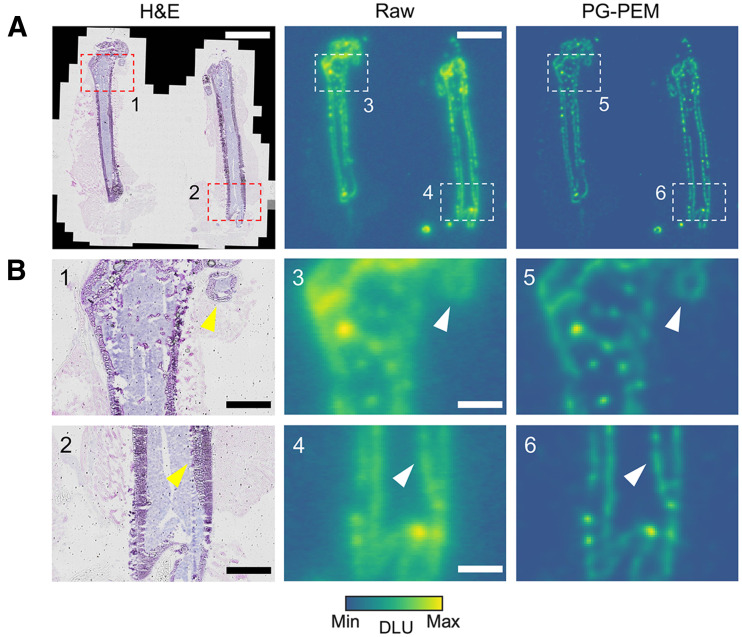
PG-PEM improves DAR images of ^18^F-NaF treated femur sections. (A) Hematoxylin- and eosin-stained, raw, and PG-PEM–restored DAR images. (B) Zoomed-in regions of corresponding boxes in A. Scale bars: 5 mm (A); 1.2 mm (B). DLU = digital light unit.

We next asked whether higher-SNR images, from ^18^F-FDG in the heart and ^18^F-NaF in the bone, could likewise be improved by PG-PEM. From the raw cardiac images, radioisotope signal is almost homogeneous. By contrast, the PG-PEM–restored data have a higher resolution and improved contrast, which may better reflect the spatial distribution of the tracer (Supplemental Figs. 13 and 15). We further compared the hematoxylin- and eosin-stained, raw, and restored DAR images of the murine femur (Fig. 5; Supplemental Fig. 16). After restoration, the endosteal and periosteal surfaces are clearly visualized, and the proximal head of the femur is resolved. Because the positron range of ^18^F is considerable, its DAR is blurred compared with lower-energy β-emitters or high-linear-energy-transfer α-emitters, hindering assessment of radiopharmaceutical distribution. Our results indicate that PG-PEM can ameliorate this issue, underscoring preclinical utility.

### Enhanced Targeted α-Particle Radiotherapy Evaluation by PG-PEM

Targeted delivery of α-particle–emitting radionuclides is an emerging application for metastatic cancer treatment (*30*,*31*). Analyzing the dose distribution for α-particle therapy near the cell scale plays a key role in predicting the treatment response and assessing the toxicity of this targeted paradigm, especially as the pathlength of α-particles is on the microscopic scale. Current small-scale dosimetry methods are based predominately on idealized computational anatomic models (*32*,*33*). Although useful, these provide limited real-world information in heterogeneous patient populations.

**FIGURE 6.**
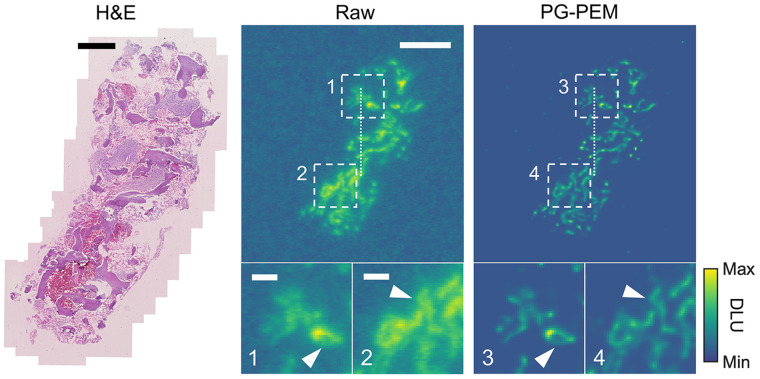
PG-PEM restoration in α-particle radiotherapy specimens. From left to right: hematoxylin- and eosin-stained histologic image of bone biopsy sample from patient with ^223^RaCl_2_-treated metastatic castration-resistant prostate cancer, and corresponding raw and PG-PEM–restored DAR images. Scale bars: 1 mm (hematoxylin and eosin); 2.3 mm (raw); 0.5 mm (insets 1 and 2). DLU = digital light unit.

We investigate α-particle emitter activity distributions from a dataset of 10 bone biopsy slides from metastatic castration-resistant prostate cancer patients treated with ^223^RaCl_2_ (Fig. 6; Supplemental Fig. 17). The raw DAR images suffer from blur and noise due to the imaging process, distorting the true radiotracer distribution. This can cause large errors in registration and degrades treatment response assessment and toxicity analysis. ^223^Ra will adsorb on the bone surface (*34*), and the high-activity regions should be located here. On the basis of this knowledge, DAR and histopathology images can be registered, and restoration algorithms can be evaluated.

**FIGURE 7.**
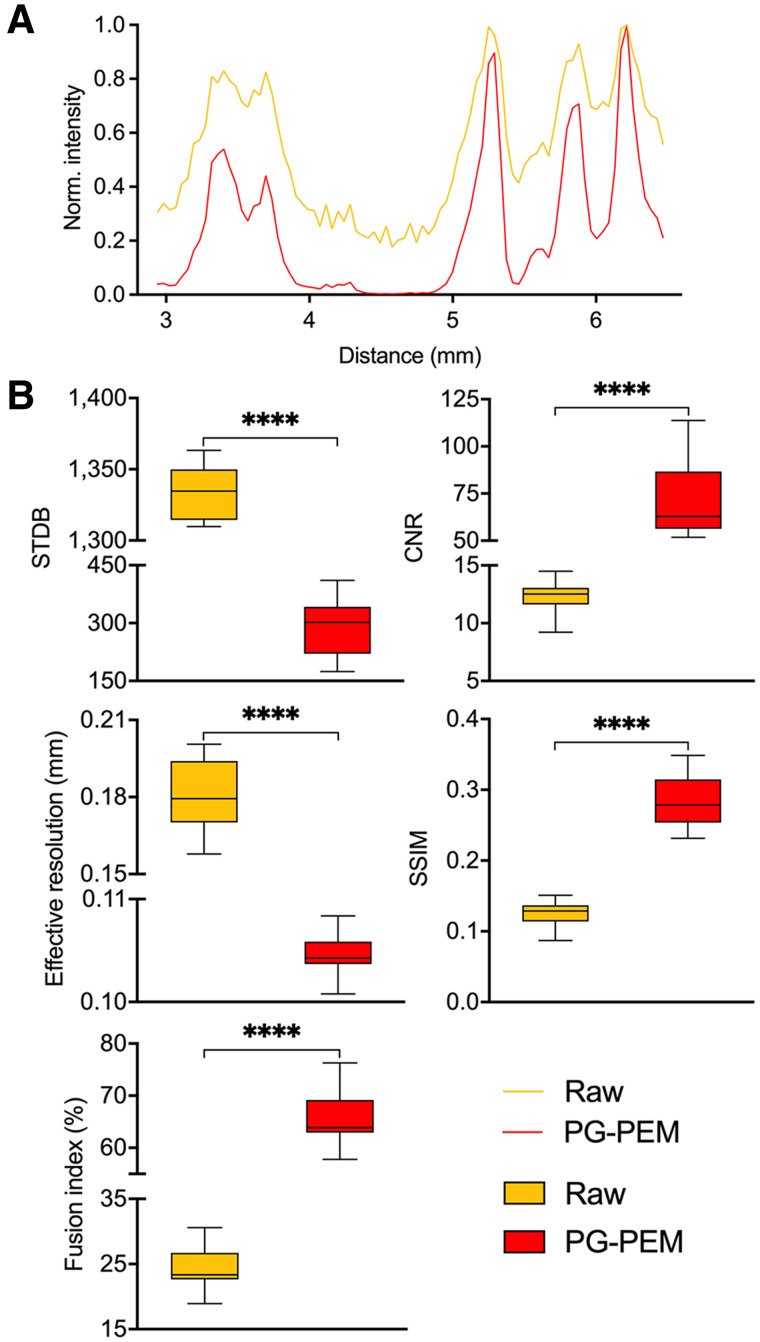
Quantitative assessment of PG-PEM for human bone biopsy DAR. (A) Profiles of dashed lines in Figure 6. (B) STDB, CNR, effective resolution, structural similarity, and fusion indices assessment for raw and restored DAR images. ****P < 0.0001.

After registration (Supplemental Fig. 18), raw and restored DAR images were fused with an anatomic bone mask (Supplemental Fig. 19). PG-PEM not only can improve the resolution and remove noise in these DAR images but also results in more accurate correlation with underlying anatomy. Quantitatively, line profiles, STDB, and CNR improve, and the effective resolution increases by approximately 1.7-fold over raw data (Fig. 7). We then calculated the structural similarity between the high-activity regions of DAR images with their segmented bone masks and evaluated a fusion index, defined as the ratio of total activity at bone surfaces (Supplemental Fig. 20). Note that the higher the structural similarity and fusion index are, the better is the correlation between the modalities. The evaluation results show that PG-PEM is able to improve these two accuracy metrics significantly (*P <* 0.0001). Consequently, PG-PEM can be of great use in personalized targeted α-particle radiotherapy assessment.

## DISCUSSION

Autoradiography is an important technique in drug development and evaluation of radiolabeled compounds for imaging and targeted therapy (*35*–*38*). In particular, there is considerable academic, pharmaceutical industry, and clinical interest in assessing targeted α- and β-particle emitters for endotherapy. Unlike external-beam radiation delivery, systemically administered radionuclides can irradiate all tissues in the body, and localized distribution is central to calculating absorbed doses and to predicting both treatment response and off-target toxicities. Conventional image formation methods using DAR suffer from noise and other image artifacts. In this work, we have defined and implemented a novel PG-PEM algorithm to restore blurred and noisy DAR data.

PG-PEM is based on the DAR imaging process and a mixed Poisson–gaussian noise model. The noise parameters are estimated with a patch-based algorithm after a Poisson–gaussian distribution conversion. A penalized MLEM approach is then used to jointly estimate the specimen image and its corresponding PSF, simultaneously. Specifically, we used the L2 norm to regularize the PSF in order to ensure its smoothness and avoid the trivial solution, and we used the Hessian Frebonius norm to regularize the estimated specimen image in order to ensure its continuity and suppress noise. Notably, this approach effectively eliminates the staircase effect caused by TV regularization. As a consequence, even low-SNR images are robustly restored. To the best of our knowledge, this is the first attempt to combine MLEM with Hessian norm–based regularization.

After analyzing the scaling factor α, we proved that it is free of precalibration in PG-PEM. Subsequently, the algorithm was quantitatively compared against alternative approaches across multiple datasets. Because of the blind restoration framework, PG-PEM is not a convex problem and we cannot guarantee that it can converge to a global solution. Nevertheless, simulation and experimental results show that PG-PEM is the lead performer, providing improved correlation between signal and tissue features.

Interestingly, even though both SP and PG-PEM are based on the mixed Poisson–gaussian noise model, PG-PEM has lower noise and reduced background false-positive signal. This difference comes from the iteration process: PG-PEM first filters gaussian-distributed noise in the expectation step and then filters Poisson-distributed noise in the maximization step. In addition, we have also compared the PSFs estimated from different isotopes (^223^RaCl_2_-treated human bone biopsy sample and ^18^F-NaF–treated mouse hindlimb). Clearly, the kernel size of the PSF from the hindlimb is larger than that from the biopsy sample (Supplemental Fig. 21), consistent with the physics of α/positron travel, further validating the blind restoration approach.

Recently, convolutional neural networks have proved effective in biomedical image restoration (*39*,*40*). However, these networks may not be well suited for DAR restoration because of multiparametric factors influencing PSF, noise characteristics for each isotope and tissue, and the lack of clean label data.

## CONCLUSION

We have developed the PG-PEM algorithm for improved DAR image quality. Predicated on a complete image formation model for DAR and implementation of a signal and background segmentation approach, this blind image restoration approach reduced background noise and image blur in simulated and primary image samples. For both high- and low-SNR datasets of diagnostic and therapeutic radionuclides, there were significant improvements in DAR resolution, contrast, and accuracy of localization. This method will be widely applicable to both preclinical- and clinical-sample autoradiograms to improve radiotracer and radiotherapy agent evaluation.

## DISCLOSURE

This work was funded in part by the National Cancer Institute of the National Institutes of Health (R01CA229893, R01CA201035, and R01CA240711 [all to Daniel Thorek]) and by the Society of Nuclear Medicine and Molecular Imaging Student Research Award (Peng Lu). No other potential conflict of interest relevant to this article was reported.

## Notes

KEY POINTS **QUESTION:** Can developments in computational imaging tools be leveraged to improve diagnostic tracer and therapeutic radionuclide distribution assessment on the tissue scale? **PERTINENT FINDINGS:** A combination of noise reduction along with automated image restoration leads to significantly enhanced DAR images. Background noise can be efficiently reduced, with improved contrast and enhanced resolution. Particular benefits are found for low-SNR images as demonstrated on clinical bone biopsies from men treated with α-particle–emitting ^223^Ra. **IMPLICATIONS FOR PATIENT CARE:** Improved understanding of radioisotope distribution on the tissue scale is expected to benefit target engagement studies for drug development and to enable more accurate dose distribution.
